# Role of Strigolactones: Signalling and Crosstalk with Other Phytohormones

**DOI:** 10.1515/biol-2020-0022

**Published:** 2020-04-10

**Authors:** Mohammad Faizan, Ahmad Faraz, Fareen Sami, Husna Siddiqui, Mohammad Yusuf, Damian Gruszka, Shamsul Hayat

**Affiliations:** 1 Tree Seed Center, College of Forest Resources and Environment, Nanjing Forestry University, Nanjing-210037, P.R. China; 2 Plant Physiology Section, Department of Botany, Aligarh Muslim University, Aligarh 202 002, India; 3 Department of Biology, United Arab Emirates University, Al-Ain, UAE; 4 Department of Genetics, Faculty of Biology and Environmental Protection, University of Silesia, Katowice, Poland

**Keywords:** arbuscular mycorrhizal fungi, carotenoids, photomorphogenesis, plant metabolism, physiological roles, seed germination

## Abstract

Plant hormones play important roles in controlling how plants grow and develop. While metabolism provides the energy needed for plant survival, hormones regulate the pace of plant growth. Strigolactones (SLs) were recently defined as new phytohormones that regulate plant metabolism and, in turn, plant growth and development. This group of phytohormones is derived from carotenoids and has been implicated in a wide range of physiological functions including regulation of plant architecture (inhibition of bud outgrowth and shoot branching), photomorphogenesis, seed germination, nodulation, and physiological reactions to abiotic factors. SLs also induce hyphal branching in germinating spores of arbuscular mycorrhizal fungi (AMF), a process that is important for initiating the connection between host plant roots and AMF. This review outlines the physiological roles of SLs and discusses the significance of interactions between SLs and other phytohormones to plant metabolic responses.

## 1 Introduction

Phytohormones are plant growth regulators synthesized within plants that participate in many aspects of the plant life cycle, including responses to biotic and abiotic stress [1, 2]. Well known examples of phytohormones include auxin, gibberellin, cytokinin, ethylene and abscisic acid (ABA): these phytohormones play important roles in plant signal transmission and they interact with each other to coordinate physiological, biochemical and morphological function in plant by establishing source/sink transition and controlling nutrient allocation [3, 4]. A variety of other compounds have more recently been recognized as important phytohormones, including a class of steroidal plant hormones called brassinosteroids (BRs), other organic molecules, such as jasmonic acid and salicylic acid and, most recently, strigolactones [5].

Strigolactones (SLs) are carotenoid derivatives that naturally occur in a variety of plants. SLs were first discovered in 1966 as root exudates in cotton plants [6]. The first identified naturally-occurring SL was “*Strigol*” [7]. SLs were known to be synthesized in roots and stems and to be transported in the xylem [8]. There are several monocots as well as dicots identified as producers of SLs, including sorghum, maize, cotton, cowpea and red clover. To date, about twenty five SLs have been extracted from different plants, of which some are *strigol, orobanchol, sorgolactone, 20-epi-orobanchol, solanacol* and *sorgomol* [9, 10]. The most common naturally-occurring SLs are characterized by a butenolide ring (D-ring) and tricyclic ring (ABC-ring), which are coupled with an enol-ether bridge in canonical SLs or to less conserved construction in non-canonical SLs [11, 12].

In 2008, SLs were categorized as a new class of plant hormone in recognition of their various roles in controlling above-ground plant architecture (e.g. by inhibiting bud outgrowth), and in underground communication with adjacent organisms [13, 14]. SLs serve dual functions, as both an endogenous and exogenous signalling molecule. Within the rhizosphere, SLs are responsible for encouraging association between plant roots and arbuscular mycorrhizal fungi (AMF), particularly in nutrient deficient environments [19]. AMF are obligate heterotrophs present in the roots of the land plants that form symbiotic associations with crop plants [15], supplying mineral nutrients in exchange for photosynthetically fixed carbon [16, 17, 18]. They also play an important role in germination of the parasitic plants *Striga* and *Orobanche* [19, 20]. Therefore, SLs serve as host detection signals for both AMF and root parasitic plants of the family *Orobanchaceae* [21]. SLs also play other important roles in the root system: enhancing the length of primary roots and root hairs, encouraging growth of rice crown roots, and suppressing the creation of adventitious roots in *Solanum lycopersicum, Arabidopsis thaliana* and *Pisum sativum* [22, 23, 24, 25, 26]. SLs are further involved in various functions related to plant growth and development such as seed germination, early seedling development, internode height, leaf structure and senescence, shoot gravitropism, and stem morphology [27, 28, 29, 30, 31, 32, 33, 34, 35]. In addition, SLs regulate the influence of environmental factors such as availability of phosphorus (P) and nitrogen (N), light intensity, drought and salinity [36, 37, 38].

This review covers what is currently known about SLs. It first outlines the many roles SLs play, including their roles in responding to plant stress. It then provides details about the biosynthesis and complex signalling mechanisms of SLs. Finally, it includes a discussion of how SLs interact (i.e. crosstalk) with other phytohormones like auxin, cytokinin and ABA.

## 2 Roles of Strigolactones

**Figure 1:**
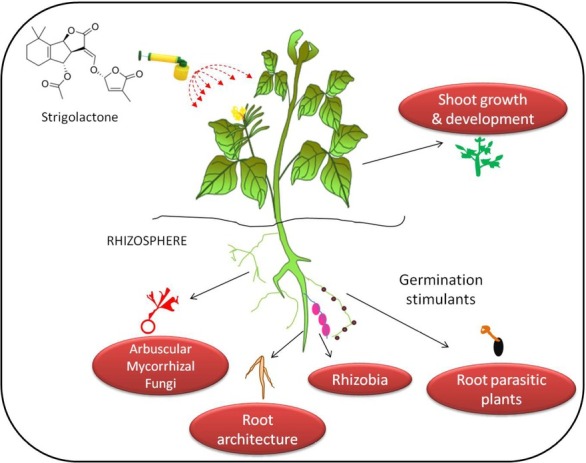
Roles of strigolactones in plant architecture.

SLs are recognised as phytohormones with roles in various developmental processes such as symbiotic mycorrhizal association between fungi and plants, plant growth, and plant development. SLs are uphill work signalling molecules that can travel from below to aboveground parts of plant and exert their effects on shoot branching control [13], host recognition signals for parasitic weeds and AMF, and responses to abiotic stress (Figure 1) [12, 14, 36, 40, 41, 42]. Some of its roles in crops plants are summarized in table 1.

**Table 1:** Known effects of SLs on plant functions in different plant species

S.N.	Plant species	Response	Reference
1	*Oryza sativa*	Enhances chlorophyll content	[29]
2	*Arabidopsis thaliana*	Represses lateral root formation	[58]
3	*Arabidopsis thaliana*	Increases resistance against abiotic stress	[70]
4	*Arabidopsis thaliana*	Promotes seed germination	[74]
5	*Arabidopsis thaliana*	Increases root hair elongation	[78]
6	*Lotus japonicus*	Delays ABA-dependent stomatal closure	[113]
7	*Pisum sativum, Arabidopsis thaliana*	Positively regulates chilling tolerance	[122]
8	*Sinorhizobium meliloti*	Enhances surface motility	[123]
9	*Oryza sativa*	Inhibits tillering trillering	[124]
10	*Bambusoideae*	Accelerates leaf senescence	[125]
11	*Sesbania cannabina*	Increases salt tolerance	[126]
12	*Solanum lycopersicum*	Plays positive role in nematode defence	[127]
13	*Glycine max*	Increases nodulation	[128]
14	*Solanum lycopersicum, Arabidopsis thaliana*	Enhances stomatal reactivity	[129]
15	*Arabidopsis thaliana*	Reduces salinity and drought stress	[130]
16	*Arabidopsis thaliana*	Increases H_2_O_2_ and nitric oxide contents	[131]
17	*Arabidopsis thaliana*	Provides resistance against bacterial infections with *Rhodococcus fascians, Pectobacterium carotovorum*, and *Pseudomonas syringae*	[132]

### 2.1 Rhizosphere and arbuscular mycorrhizal fungi

The rhizosphere is an area of soil surrounding the roots which is a very important environment for AMF [43]. AMF and plants interact with each other through signalling molecules (i.e. SLs) released by plant roots in the rhizosphere [43]. AM symbiosis is a collaborative association of plants with fungi, and is considered one of the greatest associations of plants and microorganisms. SLs play a vital role in this symbiosis [44]. SLs encourage hyphal branching in the area surrounding the host roots, which enhances the possibility of contact between the roots and fungi. Application of synthetic SLs (GR24) enhanced the colonization of mycorrhiza in petunia and pea [14, 45]. In root nodulation by rhizobia, SLs have an important role [46].

### 2.2 Shoot branching

Branching is an essential developmental process, in which axillary buds develop into flowers; it is controlled by several peripheral and internal factors that determine how energy is allocated within the plant [47]. Among the internal factors, hormones play essential roles in shoot branch management [14]. The well known phytohormones auxin and cytokinin are known to manage bud growth; cytokinin promotes growth [48] while auxin plays the role of repressor [49]. SLs are also recognised as repressors of bud growth [14]. Therefore, cytokinins act antagonistically with SLs [50]. Studies on plant architecture, using mutants, such as *decreased apical dominance* (*dad*), *high tillering dwarf* (*htd*), *more axillary growth* (*max*) and *ramosus* (*rms*) were documented in *Petunia hybrids*, *Oryza sativa*, *Arabidopsis thaliana*, and *Pisum sativum* [51]. In all these cases there is an involvement of inhibitor of branch formation [52]. Application of certain concentrations of GR24 to the *rms1* mutant plant retarded the growth of lateral bud. Similarly, exogenous application of SLs inhibited shoot branching and growth of axillary buds [41, 53].

### 2.3 Rooting and root hair

Research has shown that SLs boost elongation of primary roots and root hairs [54], but repress the formation of lateral roots [55]. *MAX2*-dependent enhancement in primary root development was noted at all concentrations of applied GR24. In lateral root formation, SLs may affect the auxin efflux by controlling the PIN proteins where auxin regulates positioning, initiation and length of lateral roots [56]. Application of SLs interferes with the PIN auxin-efflux carriers in roots and leads to reduction in the PIN1-GFP intensity in lateral root primordia, thereby altering the auxin concentration necessary for lateral root development [55]. Root hairs soak up water and nutrients from the soil, and also help in the establishment of symbiotic interactions among rhizobia and leguminous plants [57]. SL mutants of *Arabidopsis* had shorter root hairs than wild types but there were significantly longer root hairs was seen in other mutants (*max 3* and *max 4* types) and in wild plants after exogenous application of GR24 [58].

### 2.4 Leaf Senescence

Leaf senescence is an important process observed in the last stage of leaf growth [59]. It is a complex process influenced by various factors such as aging and flowering, dark treatment, nutrient starvation and several stressors [60]. During the process of leaf senescence, nutrients are reapportioned from older to younger tissue [15]. Several phytohormones are involved in the regulation of leaf senescence [61]. ABA, jasmonic acid, salicylic acid and ethylene can stimulate the process of leaf senescence, whereas cytokinins inhibit it [62]. In addition to these phytohormones, SLs also regulate the process of leaf senescence because both SL-deficient and SL-insensitive mutants show a retardation of senescence [63]. Leaf senescence is accelerated by SLs. To explore these effects on plant, GR24 was applied to the leaves of *Arabidopsis* and rice [29, 64]. Exogenous application of GR24 enhanced leaf senescence in the SL-deficient mutants of both *Arabidopsis* (*max1, max2 and max3*) and rice (*d27, d17 and d10*) [65, 66].

## 3 Strigolactones and plant stress

### 3.1 Drought

Among various abiotic stresses, drought is one of the major threats which decrease plant growth and development all over the globe [67, 68]. Similar to other phytohormones (ABA, ethylene, jasmonic acid and salicylic acid), SLs also activate signalling pathways in plants during biotic and abiotic stress conditions [69]. According to Ha et al. [70], SLs positively regulate drought response with the help of ABA signalling, as indicated by decreased sensitivity to ABA of all the *max* mutants of *Arabidopsis* under drought stress conditions. It was reported that cross-talk between SL and ABA plays an important role in integrating stress signals to regulate stomatal development and function. Both SL-deficient and SL-response mutants exhibited hypersensitivity to drought. The SL treatment rescued the drought-sensitive phenotype of the SL-deficient mutants but not of the SL-response mutant and stimulated drought tolerance of wild-type plants, confirming the role of SL as a positive regulator in the stress response [70]. Interestingly, it was reported that arbuscular mycorrhizal symbiosis induces SL biosynthesis under drought conditions and improves drought tolerance in lettuce and tomato. Under such conditions, SLs accumulation will be high that in turn interferes the organization, making it easier for plants to overcome drought stress [71]. The latest study conducted by Min et al. [72] on grapevines provides further evidence of the positive effect of SLs to overcome drought stress.

### 3.2 Temperature

Heat stress causes a variety of nutritional, hormonal, metabolic and physiological disorders that can result in crop loss [73]. Seed germination is mainly dependent on temperature; generally, high temperature slows the pace of seed germination in plants [74]. It is known that ABA is as negative regulator of seed germination while gibberellin and cytokinin play positive roles in this process [75]. SLs are positive regulators of seed germination in root parasitic weeds as well as in plants. In *Philipanche ramose*, seed germination capacity was enhanced by SL application, even at high temperature [76]. Seed germination was also enhanced by application of GR-24 under high temperature stress in SL-deficient *Arabidopsis* mutants [77].

### 3.3 Light

Light is an important factor influencing normal growth and development of plants, with modulation of light quality and quantity directly related to plant developmental processes [39]. Koltai and Kapulnik [78] found evidence of interactions between light and SLs in a variety of plants. In *Arabidopsis* plants treated with SLs (GR-24) there was increased expression of genes related to light signalling, whereas in SL-deficient plants there was low expression of those genes [79]. Light intensity modulates the levels of phytohormones, but these phytohormones also affect photoreceptor signal transduction [80]. It is suggested that higher auxin synthesis is directly related to higher production of SLs, increasing the dark-escaping phenotype. Adding evidence of a relationship between SLs and light is the fact that pea mutants produce fewer adventitious roots when compared to wild type seedlings when grown in shade but not under light conditions [15].

### 3.4 Nutrient deprivation

Availability of nutrients in the soil is very important for normal plant growth and development. Among several nutrients, Phosphorus (P) and Nitrogen (N) are essential for normal plant growth and development. SLs play a crucial role in regulating plant reactions to N and P deficiency through modification of root and/or shoot architecture and promoting symbiosis with N-fixing rhizobial bacteria and AMF [81]. P and N deficient conditions trigger increased production of SLs through an adverse feedback loop, with increased activity of SL biosynthesis genes and decreased activity of SL signalling genes [54, 82]. Under P deficient conditions plants treated with SLs form more lateral roots, whereas SL-deficient plants create fewer lateral roots. The deficiency of P and N in *Medicago truncatula* leads to over-expression of SL biosynthetic genes *CCD7*, *CCD8*, *D27* and *MAX1* [83]. Yoneyama et al. [84] selected six plant species to study the influence of P and N scarcity on biosynthesis and exudation of SLs. According to their study, there were no changes in alfalfa or tomato under N-deficient conditions, but P-deficiency enhanced the production of SLs in the remaining four plant species.

### 3.5 Biotic stress

In addition to their important functions in influencing plant architecture, SLs are important regulators of plant resistance against pathogens. Interestingly, promoters of genes involved in SL biosynthesis contain motifs which are recognised by transcription factors (TFs) involved in plant responses to pathogen infection [81]. Moreover, expression of SL-biosynthesis genes in *Arabidopsis thaliana* and *Oryza sativa* are under the control of hormones involved in plant defence such as jasmonic acid, salicylic acid and ethylene [85]. It is now known that various plant hormones, such as auxin, gibberellins, ethylene, ABA, salicylic acid, jasmonic acid, BRs and SLs, are important components of the signalling mechanisms of plant defence responses when they encounter pathogens [86]. Application of SLs strongly inhibited the formation and growth of phytopathogenic fungi [87]. SLs may overcome the stresses generated by pathogens at the transcription level with the help of the *PROTEINASE INHIBITOR II* (*PIN II*) gene [88]. It is suggested that SLs may be involved in plant immune responses in a particular bacterial and fungal pathogen-specific manner.

## 4 Signalling of SLs

**Figure 2:**
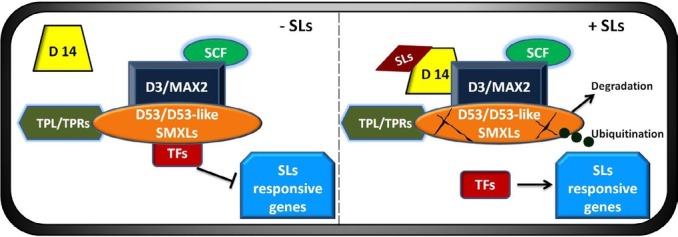
Overview of the SL signalling pathway [Modified from Wang et al. (121)]

SL signalling occurs through a precise cellular system that has been described earlier [36, 56, 89]. Based on these previous descriptions, a working model of the SL signalling pathway has been proposed (Figure 2) SLs are perceived by α/β-hydrolase that conveys the signal to a leucine-rich-repeat F-box protein (MAX2 in Arabidopsis; D3 in rice), which can bind to a Skp, Cullin, F-box (SCF)-containing complex. Such binding catalyses the ubiquitination of proteins and initiates the 26S proteasomal degradation of transcription receptors such as SMXLs in Arabidopsis and D53 in rice [90, 91, 92]. The Dwarf 14 (D14) protein is the only known receptor and is an important component of the SL signalling system [93], containing a conserved catalytic serine-histidine-aspartic acid required for hydrolytic activity [63]. This protein was initially identified in rice and later found in several other species [63, 93, 94, 95]. In the presence of SLs, D14 interacts with SLs and, through a nucleophilic attack, a D-ring derived molecule is formed which is covalently sealed in the catalytic active site of D14. This interaction triggers the conformational change of D14, leading to interaction with the D3/MAX2-based SCF complex and D53/D53-like SMXLs proteins, resulting in the degradation of proteins D53 and D53-like SMXLs through ubiquitination. This relieves the transcriptional repression on key downstream genes such as *D53* [reviewed by 36, 56, 89]. In the absence of SLs, both *D53* and D53-like SMXL proteins interrelate with TPL/ TPR proteins and suppress downstream target genes by repressing the activities of unknown transcription factors (TFs) [5, 96, 97, 98, 99, 100].

## 5 Interactions between SLs and other phytohormones

Communication and coordination among phytohormones is vital for normal plant growth. Cross-talk between hormones is controlled by special key components of signalling pathways [101]. Like other plant hormones, SLs perform several important functions through communication with other plant hormones, including auxin, GA, cytokinin and ethylene [19, 102, 103]. The sub-sections below describe research that has provided evidence of the interactions between SLs and specific other plant hormones.

### 5.1 SLs and auxin

Auxin is an important signalling molecule for plant growth and development. Auxins and SLs interact with each other in a unique feedback loop [102]. Auxins regulate SL biosynthesis and are involved in various SL-mediated developmental processes [104]. Conversely, SLs promote auxin transport in plant stems, thereby intensifying the contest between axillary branches and axillary bud outgrowth [51]. SLs play a mediator role in auxin-induced secondary messengers, moving upward to the buds and retarding their outgrowth [105]. Auxins regulate the expression of genes responsible for the synthesis of SLs [92]. However, SLs are necessary for auxin to repress decapitation-induced shoot branching [106]. According to Agusti et al. [25], SL biosynthesis and signaling is important for auxin to stimulate vascular cambium activity. SLs promote the removal rate of *PIN-FORMED1* (*PIN1*) protein, thus limiting bud outgrowth [107]. There is also evidence that auxin may interact with SLs to influence the organization of secondary growth, root development and tuberisation [108]. SLs influence and regulate auxin pathways either by facilitating auxin transport or by stimulating transcription of the auxin receptor *TIR1* [19].

### 5.2 SLs and cytokinin

Cytokinins are known to be important for a diverse range of functions in plant growth and development, influencing many agriculturally important processes [109]. SLs co-ordinate plant growth and development through cross-talk mechanisms not only with auxin and ABA, but also with other phytohormones like cytokinin and GA [19]. SLs obstruct bud outgrowth [14], while cytokinin enhances bud outgrowth. Cytokinin and SLs both act on the bud specific gene *BRANCHED 1* (*BRC1*) that encodes a transcription factor which represses bud outgrowth in *Pisum sativum* [50]. Auxins act as a secondary messenger that works in contrast to cytokinin by elevating SL levels [50]. In *Pisum sativum*, exogenous application of SLs retards axillary shoot length both under decapitation [110], or induced by cytokinin [50], adding further evidence of interactions between SLs and cytokinin.

### 5.3 SLs and abscisic acid (ABA)

**Figure 3:**
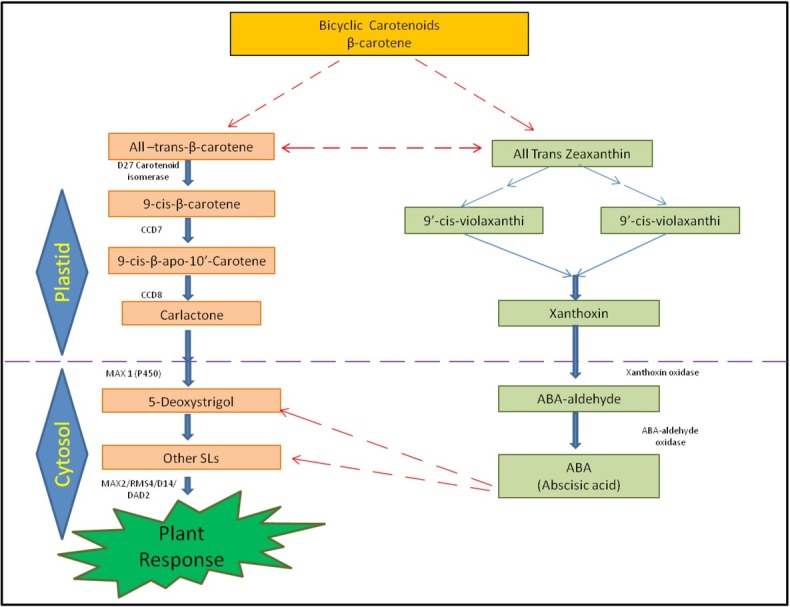
Cross-talk between SLs and ABA [Modified from Ruyter-Spira (55)]

In addition to influencing soil organisms, SLs control plant morphology and help plants respond to adverse conditions, possibly through crosstalk with ABA. SLs and ABA have similar structures, which suggest a potential connection in their biosynthesis [103]. Both SLs and ABA derive from a carotenoid pathway, in which all-trans-isomers are connected through a biosynthetic pathway of carotenoid leading from all-trans-β-carotene to all-trans-violaxanthin (Figure 3) [20]. Therefore, it is inferred that homeostasis of both hormones is mutually dependent. The isomerization activity of *9-cis/all-trans-β-carotene* of the *DWARF27* (*D27*) protein might represent possible interactions of SLs with ABA biosynthesis. Root exudates of ABA-deficient maize plants, with a null mutation in the ABA-biosynthetic gene (*ZmNCED1*), induced considerably diminished germination of parasitic seeds, and this effect is suggested to be a result of low SL content [111]. In tomatoes, reduction in ABA concentration (through chemical or genetic approaches) represses the biosynthesis of SLs [112]. Changes in the level or sensitivity to SLs influence ABA concentrations and ABA responses. The interactions between SLs and ABA are organism-dependent. SL biosynthetic mutants of *Lycopersicon esculentum* and *Lotus japonicus* are less resistant to drought stress which is also associated with elevated ABA levels in the leaves, an example of a positive interaction between the two phytohormones. Osmotic stress rapidly and sharply decreased SL content in tissues of wild-type *Lotus* roots [113] and drought stress reduced SL levels while increasing ABA content in *Lotus japonicus*, *Lycopersicon esculentum* and *Lactuca sativa* [71]. It is also known that SL metabolism and its effects on ABA homeostasis are opposite in roots and shoots under stress [113]. It is evident from the above observations that SL and ABA often fluctuate together under varied conditions with an impact on plant metabolism.

**Figure 4:**
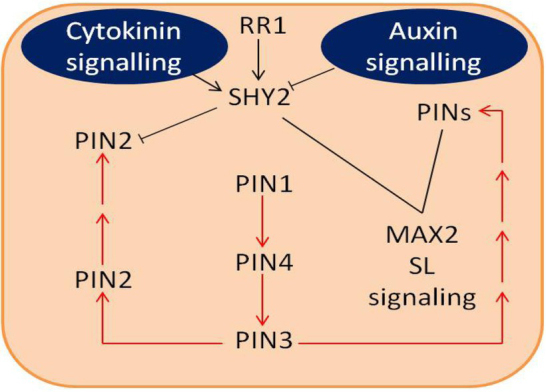
Crosstalk mechanisms between SLs, auxin and cytokinin in root tips. Brown arrows show auxin flux while black arrows and lines show assumed regulation pathways. [Figure adapted from Perilli (115), Koren (114) and Ruyter-Spira (55)]

SL signalling mechanisms may co-ordinate with auxin instability in root tips, following disruption in lateral root initiation (Figure 4). According to Koren et al. [114], SL signalling regulates auxin flux into the root tip in association with SL effects on meristem size. Furthermore, auxin and cytokinin crosstalk balances demarcation and cell partitioning in the meristem of the root tip to control root size [115]. In root apical meristem, the gene *SHY2* plays a regulatory role in determining the auxin-cytokinin balance [115]. Auxin inhibits *SHY2* activity, while cytokinin promotes the transcription of *SHY2*. The *PIN* gene is repressed by *SHY2*, which negatively effects the expression of *PIN1*, *PIN4*, *PIN3* or PIN2 transport in the root tip. Thus, cytokinin positively regulates activity of the *SHY2* gene, which enhances the flux of auxin, which ultimately affects cell division [115]. Moreover, by disturbing auxin homeostasis, *SHY2* signalling stimulates the development of existing lateral roots but inhibits their initiation [116]. Effects of SLs on lateral root initiation are mediated through auxin transport where *SHY2* is used as a molecular switch (Figure 4).

### 5.4 SLs and brassinosteroids (BR)

Crosstalk between SL and BR signalling pathways has only recently been discovered and a complete picture of this inter-hormonal crosstalk is still emerging. What is known is that this signalling interaction is mediated by crucial components of the SL and BR signalling relays. The E3 ubiquitin ligase MAX2 is a key SL signalling component, and is known to function as an inhibitor of shoot branching [80]. MAX2 directly interacts with the BZR1 and BES1 transcription factors, which are key regulators of the BR-dependent gene expression [117, 118, 119, 120]. It was reported that BZR1 and BES1 constantly interact with the MAX2 E3 ubiquitin ligase. MAX2 directly binds BZR1 and BES1, leading to degradation of these transcription factors. SL signaling promotes the MAX2-mediated degradation of the BZR1/BES1 transcription factors, and results in suppressed shoot branching. Therefore, it has been postulated that BR and SL regulate the same developmental process by modulating BZR1/BES1 stability [80].

## 6 Conclusion and future prospective

This review presents evidence that SLs are involved in a variety of processes in plant physiology and development. SLs can regulate symbiotic associations of plants with arbuscular mycorrhizal fungi, influence plant development and control plant metabolism under normal as well as stressful conditions. Only limited information is available on how SLs induce regulation of leaf senescence. However, there has been extensive research showing how important interactions between SLs and other phytohormones are for the regulation of plant growth and development. Although past research has led to considerable insight into the physiological roles of SLs, it is obvious that new functions of SLs have yet to be discovered. Interdisciplinary research is an important tool to broaden the knowledge in this area.

## Abbreviations

ABA: Abscisic acid
AMF: Arbuscular mycorrhizal fungi
BES 1: BRI1-EMSSUPPRESSOR1
BRC 1: BRANCHED 1
BRs: Brassinosteroids
BZR 1: BRASSINAZOLE RESISTANT1
CCD7: CAROTENOID CLEAVAGE DIOXYGENASE8
CCD8: CAROTENOID CLEAVAGE DIOXYGENASE8
D14: Dwarf 14
D27: DWARF27
D53: Dwarf 53
dad: Decreased apical dominance
htd: High tillering dwarf
MAX 1: More axillary growth 2
MAX 2: More axillary growth 2
N: Nitrogen
P: Phosphorus
PIN: PIN-FORMED
PIN II: PROTEINASE INHIBITOR II
rms: Ramosus
SCF: Skp-Cullin-F-box
SCF: Skp, Cullin, F-box
SHY2: SHORT HYPOCOTYL 2
SLs: Strigolactones
SMXL: SMAX1-LIKE
TFs: Transcription factors
TIR1: TRANSPORT INHIBITOR RESPONSE 1
TPL: TOPLESS
TPR: TOPLESS-RELATED
